# Gender differences in professional social responsibility: Are women more responsible at work than men?

**DOI:** 10.3389/fpsyg.2023.1049389

**Published:** 2023-01-20

**Authors:** Natalia Reig-Aleixandre, José Manuel García-Ramos, Carmen De la Calle-Maldonado

**Affiliations:** ^1^Department of Humanities, Universidad Francisco de Vitoria, Madrid, Spain; ^2^Faculty of Education, Universidad Complutense, Madrid, Spain

**Keywords:** social responsibility, gender differences, professional social responsibility, professional woman, social awareness

## Abstract

**Introduction:**

There is overwhelming evidence that companies with women on their boards of directors have higher levels of Corporate Social Responsibility. The relation between professional women and collective or organisational responsibility has been widely studied. However, to date there has been little research into the individual attitudes of women towards social responsibility. The purpose of this study is to analyse the differences in attitudes towards social responsibility between men and women in their professional life.

**Methods:**

A study sample (*N* = 524; 347 women; *M*_edad_ = 37) was assembled using the LinkedIn social media platform and participants, after providing their informed consent, were asked to answer the Professional Social Responsibility Questionnaire.

**Results:**

The results showed significant differences in Professional Social Responsibility between men and women, with moderate effect (*t*_(522)_ = 2.078; *p* = 0.038; *η*^2^ = 0.191), in favour of women. The women participants scored higher in the dimensions Discovery of Personal Values (*t*_(522)_ = 2.342; *p* = 0.020; *η*^2^ = 0.216) and Social Awareness (*t*_(522)_ = 2.179; *p* = 0.030; *η*^2^ = 0.201), both with representative effect sizes.

**Discussion:**

These results suggest that the greater commitment to Corporate Social Responsibility of companies with women on their boards of directors is due, in part, to the greater individual or personal social responsibility of women. Higher levels of Discovery of Personal Values and Social Awareness amongst women may also result in better decision-making, ultimately accruing to the benefit of the company in terms of its financial results and reputation.

## 1. Introduction

The incorporation of women into the workforce, since the second half of the 20th century, has been a social revolution with significant impacts: greater economic and social development, the strengthening of democratic values, changing family models, and, of course, greater independence and recognition of the dignity of women. There are countless studies on the incorporation of women in the workplace and its consequences. All these studies are interesting and relevant, referring to a fundamental modern socio-historical phenomenon.

The incorporation of women in the workplace has not always involved the possibility of rising to managerial roles or executive positions; after years of diligent work and progress within organisations, many women encounter the so-called “glass ceiling,” the point at which they will no longer be able to rise to positions held by the male colleagues with equal merit. Within companies there are often embedded structures that hinder the access of women to executive positions (Cortis et al., 2022).

At the same time, companies have embraced a new management model based on the Stakeholder Theory (Freeman et al., 2018) and social responsibility. Corporate Social Responsibility (CSR) is one of the key concepts in social progress and there has been a great deal of reflection within companies on the importance of Corporate Social Responsibility (Carroll, 1991; Elkington, 1997; Aguinis and Glavas, 2019; Matten and Moon, 2020).

All of this leads to the following questions: What impact has the incorporation of women had on the workplace? What has this meant for companies? and What is the relation between women and CSR?

Since 2015 there has been a proliferation of studies on the role of women on corporate boards of directors. A search using the terms *social responsibility* and *women* (title, abstract and keywords) of the Web of Sciences (WOS) and Scopus databases lists a total of 1,881 and 1,623 scientific articles, respectively, in the last 5 years (counting from June 1, 2022; Table 1).

**Table 1:** Number of articles found with the search terms *social responsibility* and *women.*

Year	No. articles in WOS	No. articles in SCOPUS
2018	319	280
2019	384	314
2020	431	369
2021	490	429
2022	257	231
Total	1881	1623

Numerous studies have found that diversity in company management not only produces more diverse opinions and better decision-making but also a clear association between the number of women in executive positions and these improved outcomes (Bernardi and Threadgill, 2011; Beji et al., 2021; Boukattaya and Omri, 2021). There appears to be an association between the number of women on the board of directors and various social behaviours such as seeking the welfare of employees, community involvement and charitable giving (Bernardi and Threadgill, 2011).

The presence of female managers on boards has a positive effect on management functions and an increase in the company’s values. This is due not only to manners of decision-making but also a greater sense of responsibility (Cook and Glass, 2018). In this sense, they help to reduce opportunistic and short-term oriented behaviour, taking a more measured view and tending towards the long term (Bashirimanesh et al., 2022).

Many of these studies suggest that improvements in CSR with the incorporation is women is largely due to their moral orientation (Cabeza-García et al., 2018). However, underlying mechanisms cannot be ruled out; for example, greater attention to the reputational standing of the company, making it more competitive in the marketplace. Women directors tend to be more interested in the reputation of the company (Hyun et al., 2016). There is also a relationship between a higher percentage of women on the board with attention to environmental measures and a higher rating of the company in this type of accreditation (Pereira, 2017).

Women executives play an important role in company management not only by promoting and implementing CSR policies but also by disseminating these policies to the public (Pucheta-Martínez et al., 2019). All of this accrues to the benefit of the company in terms of its financial results and reputation, ultimately the basis of company sustainability (Dang et al., 2021).

There seems to be a relationship between women’s participation in corporate boards and helping to combat discriminatory work environments. Social identity theory suggests that women can more easily empathise with such situations (Abebe and Dadanlar, 2021). The presence of women on the board of directors is associated with improved working conditions and human rights performance (Monteiro et al., 2022). In a study of 300 United Kingdom companies, results showed that the level of tax evasion decreases when the percentage of women on the board increases (Jarboui et al., 2020).

Amorelli and García-Sánchez (2021) conducted a bibliographical review to analyse the effect of gender diversity on corporate board meetings, in terms of company commitment to sustainability and engagement with stakeholders through the dissemination of social and environmental information. They analysed 89 articles published in 66 prestigious journals, and found that research in this area has grown spectacularly since 2016, particularly by Spanish and American researchers. The review found that the focus of studies on organisations has increasingly pivoted towards the use of Critical Mass Theory to reach conclusions about the benefits derived from having women in positions of responsibility.

Grosser (2009) addresses all these considerations and proposes to reverse the terms, namely, since women directors benefit CSR in companies, to see how CSR can also boost women’s careers.

These findings are of high interest, however, the methods used tell us only that the presence of women on boards makes the company show better CSR and improve in general. It cannot be said that the social responsibility of female directors is greater than the social responsibility of male directors, although this could be one of the causes.

The present study aims to determine if the social responsibility of women in the exercise of their profession is greater than that of their male colleagues. Our research is not limited to women in executive positions but rather will encompass all working women, regardless of their position. Nor will it be limited to the corporate world, but include all types of work; in fact, when referring to companies we use the term in the broadest sense, including small family businesses, universities, private hospitals, etc.

Our hypotheses are:

*H1:* There are significant statistical differences between men and women in their degree of Professional Social Responsibility (PSR), both globally and in each of its five dimensions, in favour of women.

*H2:* There are significant statistical differences between men and women in PSR, according to professional position.

*H3:* There are significant statistical differences between men and women in PSR, according to age.

*H4:* There are significant statistical differences between men and women in PSR, according to company size.

*H5:* There are significant statistical differences between men and women in PSR, according to previous studies.

## 2. Methods

The study used a descriptive, ex post-facto and correlational design to analyse if there are significant differences between the group of women (key group) and the group of men (quasi-control group; León and Montero, 2020).

### 2.1. Variables

The independent variable (IV) was gender, and the dependent variable was the degree of PSR and each of its 5 dimensions. The study also took into consideration the following secondary independent variables: age, education, type of work and position in the company.

### 2.2. Participants

The population consists of the active population of the Community of Madrid (Spain). A minimum sample size of 385 subjects was determined by the IT program ENE 3.0 for a finite sample, with a precision of 0.50 and a confidence level of 0.95. An incidental, “snowball” sample was created using the social network site LinkedIn. This social network largely reflects the professional life of users and the majority, although not all, have a university education. We contacted possible candidates and explained the purpose of the study. After providing their informed consent participants were asked to complete the questionnaire. A total of 524 subjects participated in the study, 347 women (66%) and 177 men (34%) with an average age of 37.

Only 16.1% of the women in the sample were executives (Table 2). We are also interested in the professional social responsibility of the remaining 83.9%.

**Table 2:** Research sample by groups of the IV and position in the company.

Gender			Woman	Man	Total
Position	Technical staff	Count	152	60	212
		% by gender	43.8%	33.9%	40.5%
		% del total	29.0%	11.5%	40.5%
	Middle Management	Count	139	69	208
		% by gender	40.1%	39.0%	39.7%
		% del total	26.5%	13.2%	39.7%
	Executive	Count	56	48	104
		% by gender	16.1%	27.1%	19.8%
		% del total	10.7%	9.2%	19.8%
Total		Count	347	177	524
		% del total	66,2%	33.8%	100.0%

### 2.3. Measurement

Socio-demographic data. Participants were asked to complete a questionnaire in which they were asked: gender, age, education, type of work, position in the company.

Professional Social Responsibility Questionnaire (Reig-Aleixandre, 2020). This instrument consists of 31 items, 30 related to PSR behaviour and 1 item concerning a global evaluation of PSR behaviour. The questionnaire used a Likert-type scale from 1 to 6, with 1 meaning “never” or “nothing” and 6 meaning “always” or “all.” Some examples of the questions are: “I believe it is important to have ethical values and to try always to remain faithful to them”: “I optimise the use of resources in my way of working.” The reliability of the overall scale (*α* = 0.99) and its 5 dimensions (*α* = 0.77; 0.74; 0.87; 0.81; 0.81 respectively) obtained adequate values. The composite reliability of the construct, both total scale (*ω* = 0.93) and of the dimensions (*ω* = 0.77; 0.73; 0.87; 0.77; 0.81 respectively) was adequate. The Convergent Criterion Validity was found to be adequate both in the Scale (*r*_(522)_ = 0.67; *p* = 0.01) and in the 5 Dimensions (*r*_(522)_ = 0.47; 0.66; 0.53; 0.54; 0.61 respectively). The exploratory factor analyses (EFA) and confirmatory factor analyses (CFA) generally confirmed the structure of the measurement instrument and is correspondence with the PSR Construct. With respect to the EFA, both the KMO test (0.940) and Bartlett’s test of sphericity (*p* < 0.0001) showed that the correlation matrix was adequate for the analysis. On the other hand, the CFA yielded good indices of model fit to the data obtained (*χ*^2^/gl = 3.15; GFI = 0.93; CFI = 0.93; TLI = 0.92; RMSEA = 0.06; SRMR = 040).

### 2.4. Data analysis

To test Hypothesis 1, in addition to the main descriptive statistics, Student’s *t*-test and Cohen’s *d* test were used. In order to verify Hypotheses 2–5, F factorial ANOVA was used, taking the RSP score as the dependent variable and gender, position, age, company size and previous studies as independent variables. In this statistical test, the interactions of the gender variable with the rest of the independent variables were included. Effect sizes were also found using the Eta^2^ statistic. IBM SPSS version 25 software was used to analyse the data.

The professional social responsibility questionnaire had already been validated in another study. However, the composite reliability was tested using McDonald’s *ω* statistic. This was done using IBM SPSS software version 29.

## 3. Results

### 3.1. Hypothesis 1: Statistical differences between men and women in their degree of Professional Social Responsibility

Regarding Global Professional Social Responsibility (PSR), the Student’s *t*-test showed significant results (*t*_(522)_ = 2.078; *p* = 0.038; *d* = 0.19) with a moderate effect, in favour of women (*M*: 155.78; SD: 18.12) compared to men (*M*: 152.25; SD: 18.87). For Dimension 1 (Discovery of Personal Values), a significant and representative result was observed (*t*_(522)_ = 2.342; *p* = 0.020; *d* = 0.22). Here the results were also in favour of women (*M*: 32.79; SD: 3.61) compared to men (*M*: 32.03; SD: 3.29). Finally, in Dimension 2 (Social Awareness) there was also a significant and representative result (*t*_(522)_ = 2.179; *p* = 0.030; *d* = 0.20) in favour of women (Tables 3, 4).

**Table 3:** Descriptive statistics.

Dimensions	Gender	*N*	Mean	SD
1. Discovery of personal values	Woman	347	32.79	3.61
	Man	177	32.03	3.29
2. Social awareness	Woman	347	30.91	4.04
	Man	177	30.07	4.42
3. Commitment to others	Woman	347	31.78	4.51
	Man	177	31.39	4.51
4. Commitment to the environment	Woman	347	28.96	5.24
	Man	177	28.18	5.33
5. Consideration of profession as service	Woman	347	31.35	4.60
	Man	177	30.59	4.76
Total PSR scale	Woman	347	155.78	18.12
	Man	177	152.25	18.87

**Table 4:** Student’s *t* test for independent samples.

Dimensions	*t*	df	Value of *p*	Cohen’s *d*
1. Discovery of personal values	2.342	522	0.020	0.216
2. Social awareness	2.179	522	0.030	0.201
3. Commitment to others	0.932	522	0.352	–
4. Commitment to the environment	1.606	522	0.109	–
5. Consideration of profession as Service	1.763	522	0.079	–
Global PSR scale	2.078	522	0.038	0.192

However, no significant differences were found in Dimension 3 (*p* = 0.352), Dimension 4 (*p* = 0.109) or Dimension 5 (*p* = 0.079; Table 4).

### 3.2. Hypothesis 2: Statistical differences in PSR, according to professional position

No significant differences were found between men and women according to their position in the company (*p* = 0.126). An analysis was made by groups; no significant differences were observed between the groups of men (*p* = 0.631) in Global PSR or any of the dimensions. For groups of women no significant differences were found for Global PSR (*p* = 0.062). However, significant differences were observed (*F*_(2.346)_ = 3.647; *p* = 0.027) amongst women in Dimension 3 (Commitment to Others). Middle managers (*M*: 32.12; SD: 3.59) scored higher than technical staff (*M*: 32.01; SD: 4.00) and executives (*M*: 30.30; SD: 7.01).

There were also significant differences (*F*_(2.346)_ = 3.924; *p* = 0.021) in Dimension 4 (Commitment to the Environment) where middle managers (*M*: 29.87; SD: 5.02) scored higher than technical staff (*M*: 28.53; SD: 5.59) and executives (*M*: 27.86; SD: 4.46). These results were confirmed in a post-hoc analysis.

### 3.3. Hypothesis 3: Statistical differences in PSR, according to age

Significant differences were found between men and women according to age, in Global PSR, in Dimension 1, Dimension 2 and Dimension 5. In all these cases the effect size was weak. A factor analysis including a second independent variable (age) showed that only the gender variable accounted for significant differences. These same differences were found in the Student’s *t*-test. However, results for Dimension 5 were also significant (*F*_(1.516)_ = 5.18; *p* = 0.23; *η*^2^ = 0.010), due to a lower error variance in the analysis (Tables 5, 6).

**Table 5:** Gender differences in the factorial ANOVA test gender-age.

Dimensions	*F*	Value of *p*	*η* ^2^
1. Discovery of personal values	5.20	0.023	0.010
2. Social awareness	6.49	0.011	0.012
3. Commitment to others	3.287	0.070	–
4. Commitment to the environment	3.170	0.076	–
5. Consideration of profession as service	5.18	0.023	0.010
Global PSR scale	6.479	0.011	0.012

**Table 6:** Descriptive analysis of the group of women according to age.

Dimensions	Age	Mean	SD	*N*
Global PSR	<30	155.02	13.47	127
	From 30 to 34	158.56	13.20	50
	From 35 to 39	156.91	12.95	32
	Over 39	155.21	23.63	138
	Total	155.78	18.12	347
1 Dimension: Discovery of Personal Values	<30	32.54	2.88	127
	From 30 to 34	33.66	2.02	50
	From 35 to 39	32.66	2.51	32
	Over 39	32.73	4.70	138
	Total	32.79	3.61	347
2 Dimension: Social Awareness	<30	30.60	3.63	127
	From 30 to 34	31.40	3.15	50
	From 35 to 39	30.97	2.91	32
	Over 39	31.01	4.85	138
	Total	30.91	4.04	347
5 Dimension: Consideration of Profession as Service	<30	31.48	3.67	127
	From 30 to 34	31.58	3.95	50
	From 35 to 39	31.34	3.39	32
	Over 39	31.14	5.74	138
	Total	31.35	4.60	347

As for mean differences, the group with the highest scores for Global PSR (*M*: 158.56; SD: 13.20) were those aged 30–34; those with the lowest scores for PSR (*M*: 155.02; SD: 13.47) were those under 30 (Table 6).

### 3.4. Hypothesis 4: Statistical differences in PSR, according to company size

No significant differences were found for PSR between men and women according to size of the company. Results for Global PSR were not significant (*p* = 0.192) nor were those in the other dimensions (*p* = 0.098; 0.164; 0.463; 0.435 and 0.293, respectively).

### 3.5. Hypothesis 5: Statistical differences in PSR, according to previous studies

Significant differences were found between men and women according to education for Global PSR, Dimension 1 and Dimension 2. In all cases the effect size was weak (Table 7).

**Table 7:** Gender differences in the factorial ANOVA test gender-education.

Dimensions	*F*	Value of *p*	*η* ^2^
1. Discovery of personal values	6.885	0.009	0.013
2. Social awareness	5.849	0.016	0.011
3. Commitment to others	1.555	0.213	–
4. Commitment to the environment	2.36	0.125	–
5. Consideration of profession as service	2.935	0.087	–
Global PSR scale	4.950	0.027	0.009

The group of women with the highest scores in Global PSR were those with a degree in Experimental Sciences (*M*: 160.00; SD: 14.40). The group of women with the lowest scores for Global PSR were those with a degree in Humanities and/or Education (*M*: 154.10; SD: 23.26; Table 8).

**Table 8:** Descriptive analysis of the group of women according to university education.

Dimensions		Mean	SD	*N*
Global PSR	Business and/or Law	156.46	20.86	63
	Health sciences	155.90	16.47	73
	Experimental sciences	160.00	14.40	44
	Communications	154.41	13.48	61
	Engineering	155.08	10.83	25
	Humanities and/or Education	154.10	23.26	81
	Total	155.78	18.12	347
1 Dimension: Discovery of personal values	Business and/or Law	33.03	4.21	63
	Health sciences	33.08	2.82	73
	Experimental sciences	33.64	1.98	44
	Communications	32.82	3.09	61
	Engineering	32.60	2.04	25
	Humanities and/or Education	31.90	4.86	81
	Total	32.79	3.61	347
2 Dimension: Social awareness	Business and/or Law	31.37	4.62	63
	Health sciences	31.08	4.14	73
	Experimental sciences	31.18	3.15	44
	Communications	30.69	3.53	61
	Engineering	30.84	2.44	25
	Humanities and/or Education	30.46	4.66	81
	Total	30.91	4.04	347

## 4. Discussion

After several decades of women’s entry into the workforce, the question arises as to what contribution they are making to the company and, in general, to the professional sphere. Many studies show that women’s participation on boards of directors significantly improves the company’s CSR. However, we wonder whether this improvement is due to the fact that women are more socially responsible in the exercise of their profession. The results of this research aim to provide an answer to this question.

Firstly, the study’s findings are highly important as they show greater commitment to CSR amongst companies in which women hold executive positions. This may be due to the greater dedication to Professional Social Responsibility (PSR) amongst women (Hypothesis 1: There are significant statistical differences between men and women in their degree of PSR). Women also score higher in Discovery of Personal Values, which may explain better decision-making (Boukattaya and Omri, 2021) and, of course, the increase in the company’s values (Bashirimanesh et al., 2022). This greater appreciation of values could also be related to a decrease in discriminatory work environments (Abebe and Dadanlar, 2021) and improved working conditions in general (Monteiro et al., 2022). In addition, they also show greater social awareness, which could be related to a decrease in tax evasion when there are more women on the board (Jarboui et al., 2020). This higher Social Awareness score could also be associated with the greater promotion and implementation of CSR policies by women, as noted by Dang et al. (2021), ultimately accruing to the benefit of the company in terms of its financial results and reputation. Therefore, this confirms that when women join boards of directors, they do not improve their decisions only because of the diversity factor, but because they bring social awareness, concern for values and, ultimately, a sense of social responsibility (Bernardi and Threadgill, 2011; Beji et al., 2021; Boukattaya and Omri, 2021).

Furthermore, it is revealing that women in executive position show lower levels of Professional Social Responsibility than women in middle management and technical staff (Hypothesis 2: There are significant statistical differences between men and women in PSR, according to professional position). To date, studies have focussed on the impact of women on corporate boards of directors. This is understandable given the importance of those in executive positions, both for the company and for society. However, considering these results, it would be highly interesting to further explore the consequences of PSR amongst women, regardless of their position in the company.

In this line, the study found that women over the age of 39 have lower levels of PSR than those aged 30–34 and 34–39 (Hypothesis 3: There are significant statistical differences between men and women in PSR, according to age). There is a generally held belief that people become more responsible as they get older and these results were surprising and invite further study.

There are significant differences in gender according to previous studies. Women who studied Experimental Sciences and Business and/or Law showed higher scores, whilst women who studied Humanities and/or Education scored lower (Hypothesis 5: There are significant statistical differences between men and women in PSR, according to previous studies). It may be asked to what extent these differences are due to the differing personalities of those studying these degrees or pursuing university education more generally.

Secondly, it would be very interesting to compare the results of the present research with those of other similar studies but, as noted above, as yet we have found no studies into the differences in PSR between men and women. However, there have been studies into the attitudes of university students towards social responsibility. A study by Bustamante and Navarro (2007) of students of Social Sciences in Chile found significant differences between men and women, with women self-reporting higher levels of socially responsible behaviour. Villa and Villa (2007) analysed the perceived importance ascribed to various competences by recent graduates from the Universidad de Deusto (Spain). Reig-Aleixandre et al. (2021) conducted research with a sample of 612 young people from 7 Spanish universities and also found a significant difference in the degree of social responsibility in favour of women. The results showed that women gave greater value to social commitment, coherence with one’s values and civic spirit than men. Severino-González et al. (2019) analysed the social responsibility of Chilean university students and found significant differences in favour of women in their commitment to the environment and in actions linked to sustainable development. The results of the present study are not comparable given that, firstly, the study analysed Social Responsibility in professional practice; secondly, the sample populations are different. This study focuses on the adult population. Nevertheless, it is interesting to note the significant differences in attitudes towards social responsibility are always favourable to women.

## 5. Conclusion

The incorporation of women into the workplace, and especially in executive positions, has afforded several benefits for both companies and society, including the promotion and implementation of social responsibility initiatives. Studies to date have largely focussed on the relation between having women present on boards of directors and corporate outcomes. This study analysed the possible relation between this improvement in Corporate Social Responsibility and the personal attitudes towards social responsibility amongst women. The findings, based on an adequate and representative sample, show that women have greater Professional Social Responsibility than their male counterparts, are unprecedented. However, given the novelty of the research topic, it would be useful to corroborate the results in other samples with different characteristics, larger in size and not accessed through the LinkedIn social network. An important limitation of this study was accessing the sample through this social network. The users are mainly young and middle-aged, which lowers the average age (37 years) and, on the other hand, many of them are university graduates. For future research it would be necessary to access the sample in other ways to reach older workers and those without university education. Hypotheses 2, 3, and 5 (that is differences according to professional position, age and company size, respectively) of this study also present interesting prospects that we have not been able to address sufficiently and would need to do so in future research.

## Data availability statement

The original contributions presented in the study are included in the article/supplementary material, further inquiries can be directed to the corresponding author.

## Author contributions

NR-A wrote the manuscript, collected the data and analysed it. JG-R performed and assisted in the statistical analyses and reviewed the results. CC-M collaborated in writing the manuscript and reviewed the discussion. All authors contributed to the article and approved the submitted version.

## Funding

This research has been funded by the Faculty of Education and Psychology (Universidad Francisco de Vitoria); by the Faculty of Law, Business and Government (Universidad Francisco de Vitoria); and by the Research Project UFV2021-53 (Universidad Francisco de Vitoria).

## Conflict of interest

The authors declare that the research was conducted in the absence of any commercial or financial relationships that could be construed as a potential conflict of interest.

## Publisher’s note

All claims expressed in this article are solely those of the authors and do not necessarily represent those of their affiliated organizations, or those of the publisher, the editors and the reviewers. Any product that may be evaluated in this article, or claim that may be made by its manufacturer, is not guaranteed or endorsed by the publisher.
